# Editorial: Spirochetal diseases (syphilis, Lyme disease, and leptospirosis): transmission, pathogenesis, host-pathogen interactions, prevention, and treatment

**DOI:** 10.3389/fmicb.2024.1510000

**Published:** 2024-10-30

**Authors:** Christopher J. Pappas, Camila Hamond, Helena Pětrošová, Ellie J. Putz

**Affiliations:** ^1^Division of Natural Sciences, Mathematics, and Computing, Manhattanville University, Purchase, NY, United States; ^2^Department of Pathobiology and Veterinary Science, College of Agriculture, Health and Natural Resources, University of Connecticut, Storrs, CT, United States; ^3^National Reference Laboratory for Leptospirosis, World Health Organization Collaborating Center for Leptospirosis, Instituto Oswaldo Cruz, Rio de Janeiro, Brazil; ^4^University of Victoria Genome BC Proteomics Centre, Victoria, BC, Canada; ^5^Department of Biochemistry and Microbiology, University of Victoria, Victoria, BC, Canada; ^6^National Animal Disease Center, Agricultural Research Service (USDA), Ames, IA, United States

**Keywords:** leptospirosis, Weil's disease, syphilis, Lyme disease, treatment, host-pathogen interactions, diagnosis, immune response

Order Spirochaetales are Gram-negative and Gram-negative-like bacteria with unique morphological and functional features. They are distinguished from other bacteria by the presence of endoflagella, which gives this Phylum of bacteria spiral morphology and distinct motility (San Martin et al., 2023). Their unusual cellular ultrastructure, motility, and metabolic pathways, immune evasion strategies, and gene regulation has evoked the maxim “spirochetes do it differently” (Charon et al., 2012). Within the Phylum, there are several orders of spirochetes of importance to human health. These comprise Leptospirales and Spirochetales. The leptospires include the Genus *Leptospira* which have distinct pathogenic potential, comprising infectious and free-living non-infectious species. Pathogenic species (e.g., *L. interrogans* and *L. borgpetersenii*) are the causative agents of the disease leptospirosis, which can manifest as fever, kidney and liver dysfunction, and Weil's disease, among other sequelae (Adler and de la Pena Moctezuma, 2010). Within Spirochetales are two Genera of importance to human health: *Treponema* and *Borrelia*. *Treponema* include several species of medical importance, including *Treponema pallidum* subspecies *pallidum*, which is the etiologic agent of syphilis (Norris et al., 2015). In *Borrelia*, several species have been identified as the causative organisms of Lyme disease, including *Borrelia burgdorferi* and *Borrelia garinii* (Steere et al., 2016). Spirochetal diseases pose immense and growing global threats to human and animal health, as well as an economic burden to impacted communities.

This Research Topic has been organized to better understand the pathogenesis and escape mechanisms of spirochetes, along with host-pathogen interactions, immune evasion mechanisms, prevention strategies, and novel treatment strategies for spirochetal infections. Highlights from the Research Topic's manuscripts are summarized below.

One of the areas of concern is accurate diagnosis of an active infection in people with suspected syphilis, as the current tests have high false positive and false negative rates. Silva et al. determined the efficacy of using two *T. pallidum* proteins as serological markers of an active infection by indirect ELISA in various stages of disease. Their findings demonstrated that using TpN17 along with TmpA had high sensitivity and specificity for detection of *T. pallidum* infection, with minimal cross-reactivity with other pathologies. Another area of consideration with diagnoses is cost-effectiveness. In the study by Zhang et al. the authors conducted a literature review to assess the cost-effectiveness of screening for syphilis in pregnant women. The authors concluded that early stage screening is not only cost effective, but also increases the likelihood of positive outcomes for both the mother and the fetus.

Once spirochetes enter into a mammalian host, they undergo various gene expression changes to adapt to their new physiological environment. At the same time, these pathogens can alter the host environment to disseminate and establish infection. Waugh et al. described how *T. pallidum* alters host pathways in endothelial cells, including changes to the extracellular matrix milieu, innate immune signaling, and cytokine response within host cells. Shen et al. demonstrated by flow cytometry that the total number of T follicular helper cells varies in percentage and type by stage of syphilis. In *Borrelia*, VLS and VLS-like proteins, which are important for immune evasion in *Borrelia* species, were reanalyzed by Norris and Brangulis to decipher gene conservation and divergence amongst various *Borrelia*. Finally, the review by Surdel and Coburn described how pathogenic *Leptospira* adhere to host molecules. They also discuss current methodologies used to study adhesins, and conclude with future avenues of research and the potential for anti-adhesion therapies.

Finally, research into treatment methodologies and prognosis of spirochetal infections is important to human health. In the article, by Wu et al. the authors ran a retrospective case control study on 86 HIV and syphilis co-infected patients and found elevated cases of syphilis reinfection in patients that were effectively treated for the disease. They concluded that education of patients along with follow-up care could help these patients avoid reinfection with *T. pallidum*.

With an increase in global temperature due to climate change, it is anticipated that cases of leptospirosis and Lyme disease will increase in the coming years (Lau et al., 2010; Beard et al., 2016). Syphilis is sometimes considered a disease of the past, but its incidence has been sharply rising globally. This alarming increase, coupled with the worldwide shortage of the first-choice antibiotic treatment benzathine penicillin G, underscores the urgent need for increased surveillance, novel laboratory testing, and treatment strategies (Valentine and Bolan, 2018). Collectively, these exceptional manuscripts aid our understanding of these diseases and their underlying etiologic agents, and help us toward the path of disease control.
